# Complex regulation of γ-secretase: from obligatory to modulatory subunits

**DOI:** 10.3389/fnagi.2014.00342

**Published:** 2015-01-06

**Authors:** Natalya Gertsik, Danica Chiu, Yue-Ming Li

**Affiliations:** ^1^Molecular Pharmacology and Chemistry Program, Memorial Sloan-Kettering Cancer CenterNew York, NY, USA; ^2^Biochemistry and Molecular Biology Program, Weill Graduate School of Medical Sciences of Cornell UniversityNew York, NY, USA; ^3^Program of Pharmacology, Weill Graduate School of Medical Sciences of Cornell UniversityNew York, NY, USA

**Keywords:** γ-Secretase, Alzheimer’s disease, presenilin, β-amyloid, Notch, APP, Hif-1α

## Abstract

γ-Secretase is a four subunit, 19-pass transmembrane enzyme that cleaves amyloid precursor protein (APP), catalyzing the formation of amyloid beta (Aβ) peptides that form amyloid plaques, which contribute to Alzheimer’s disease (AD) pathogenesis. γ-Secretase also cleaves Notch, among many other type I transmembrane substrates. Despite its seemingly promiscuous enzymatic capacity, γ-secretase activity is tightly regulated. This regulation is a function of many cellular entities, including but not limited to the essential γ-secretase subunits, nonessential (modulatory) subunits, and γ-secretase substrates. Regulation is also accomplished by an array of cellular events, such as presenilin (active subunit of γ-secretase) endoproteolysis and hypoxia. In this review we discuss how γ-secretase is regulated with the hope that an advanced understanding of these mechanisms will aid in the development of effective therapeutics for γ-secretase-associated diseases like AD and Notch-addicted cancer.

## Introduction

**Figure 1:**
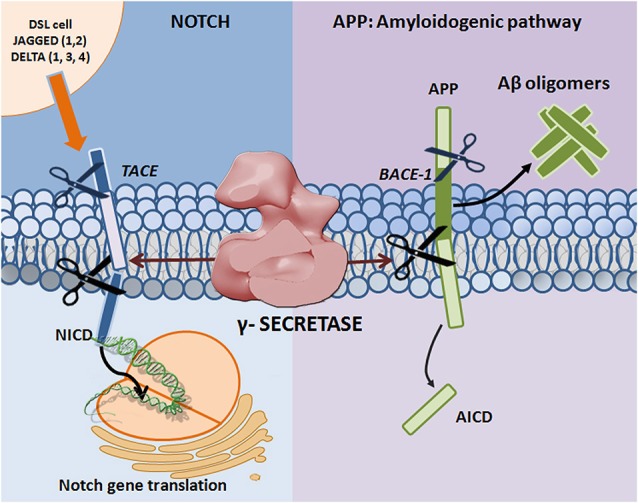
**Proteolytic processing of APP and Notch**. Mature Notch receptors are activated by binding to ligands (Jagged-1, -2 and Delta-like -1, -3, and -4) located on adjacent signal-presenting cells. An induced conformational change exposes a cleavage site (S2) for ADAM family metalloproteases that cleave Notch at an extracellular, membrane-proximal region. The membrane-bound Notch segment that results from this cleavage, known as Notch Intracellular Truncation domain (NEXT), is a γ-secretase substrate (Kopan and Ilagan, 2009). γ-Secretase performs the subsequent cleavage at S3 (De Strooper et al., 1999), releasing Notch intracellular domain (NICD) from the membrane and allowing for signal transduction through binding with the CBL-1, Su(H), Lag-1 (CSL; Schroeter et al., 1998; Struhl and Adachi, 1998) family of DNA binding proteins. APP undergoes sequential proteolytic processing first by β-secretase (BACE1, aspartyl protease) and then by γ-secretase, in the amyloidogenic pathway. The first cleavage results in ectodomain shedding in which the amino-terminal of APP is removed, yielding a soluble APP derivative (sAPPβ) and a carboxy-terminal membrane stub known as βCTF (C99). βCTF is a substrate for γ-secretase, and is cleaved in its transmembrane domain to form AICD and the potentially toxic Aβ. Mutations in presenilin (the catalytic subunit of γ-secretase) and APP can lead to increases in the Aβ42 to Aβ40 ratio, resulting in Aβ deposition and plaque formation.

γ-Secretase is an intramembrane aspartyl protease that cleaves an array of type 1 transmembrane substrates, of which amyloid precursor protein (APP) and Notch are the most widely studied. APP undergoes sequential proteolytic processing by β-secretase (BACE1) and γ-secretase to generate amyloid beta (Aβ) peptides, which are 37–43 amino acids long. Notch, a protein that resides on the surface of signal-receiving cells as a heterodimeric receptor, is also subject to a series of proteolytic cleavages (Figure 1). The scientific scrutiny sustained by both APP and Notch results from their role in disease: aberrant γ-secretase cleavage of APP and Notch can lead to Alzheimer’s disease (AD) and cancer, respectively. γ-Secretase is an important potential drug target for both diseases and γ-secretase inhibitors (GSIs) and modulators (GSMs) are currently in clinical trials.

**Figure 2:**
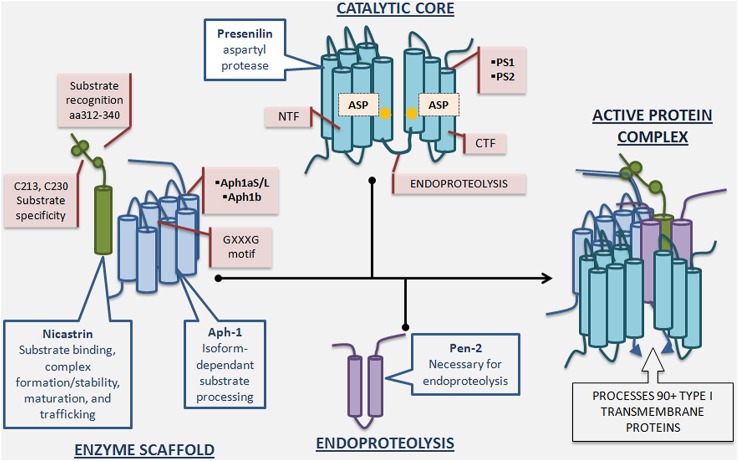
**γ-Secretase complex formation and regulatory roles of individual essential subunits**. The γ-Secretase complex is formed by the sequential assembly of Aph1, nicastrin, presenilin, and Pen-2. First, Aph-1 and nicastrin come together to form the scaffold. Next, full length presenilin is incorporated. Last, Pen-2 is recruited and full length presenilin is endoproteolysed into presenilin-NTF/CTF, activating the enzyme. Nicastrin, a heavily glycosylated single-pass transmembrane protein, plays a role in scaffolding, enzyme stabilization, substrate recognition, and trafficking. Nicastrin’s amino acids 312–340 are important for substrate recognition and deletion of these residues reduces γ-secretase activity and nicastrin’s interaction with APP and Notch. Furthermore, mutation of nicastrin’s C213 and C230 leads to different impact on processing of APP and Notch, underscoring nicastrin’s role in substrate selectivity. Aph-1, a 7-pass transmembrane protein with 3 human isoforms, is crucial for scaffolding and stability, and may have an additional role in determining length of Aβ species produced depending on which isoform is incorporated into the γ-secretase complex. The GXXXG motif in Aph-1 is critical for γ-secretase complex assembly. Presenilin, a 9-pass transmembrane protein with 2 isoforms, is the catalytic subunit of γ-secretase, and full length presenilin is a zymogen that must be endoproteolysed into NTF/CTF to be enzymatically active. Mutations in presenilin1 and presenilin2-encoding genes account for the majority of genetic mutations leading to Familial Alzheimer’s disease. Pen-2, a 2-pass transmembrane protein, is required for presenilin endoproteolysis and γ-secretase activation, but also may play an endoproteolysis-independent role in γ-secretase regulation. Active γ-secretase constitutes a small percentage of total γ-secretase and resides primarily in the plasma membrane.

Biochemical studies indicated that γ-secretase activity is catalyzed by the presenilin (PS)-containing macromolecular complex (Li et al., 2000a). The search for other components of the complex revealed three additional proteins: nicastrin (Nct), anterior pharynx-defective-1 (Aph-1), and presenilin enhancer-2 (Pen-2) (Yu et al., 2000; Francis et al., 2002; Goutte et al., 2002). It has since been established that these four proteins constitute the mature γ-secretase complex (De Strooper, 2003; Selkoe and Wolfe, 2007), and their stepwise assembly, followed by endoproteolysis of PS into amino-terminal (PS-NTF)and carboxy-terminal fragments (PS-CTF), is necessary for active complex formation (Takasugi et al., 2003) (Figure 2). The recent report of a 4.5Å cryo-electron microscopy structure of intact human γ-secretase and identification of novel γ-secretase modulating mechanisms have provided insight into the flexibility and complexity of this enzyme (Lu et al., 2014).

γ-Secretase processing of its substrates produces distinct amino and carboxy termini with variable functions; some products of γ-secretase cleavage function as transcriptional regulators while others are thought to play roles in signaling, cell adhesion, and cytoskeletal dynamics. As the list of putative γ-secretase substrates continues to grow, now reaching over 90 reported proteins (Haapasalo and Kovacs, 2011), the lack of homology between these substrates becomes increasingly apparent: other than the fact that they are all type I transmembrane proteins that have undergone ectodomain shedding, γ-secretase substrates are surprisingly dissimilar (Beel and Sanders, 2008; Lleó, 2008) (of note, how many of these substrates are actually processed by γ-secretase *in vivo* remains to be investigated). Not only are the substrates themselves widely variable, but cleavage of some substrates (i.e., CD44 and Notch-1) (Lammich et al., 2002; Okochi et al., 2002) leads to release of peptides with variable carboxy-terminal ends, further confirming γ-secretase’s astounding promiscuity. In short, γ-secretase not only cleaves many substrates, but it cleaves the same substrate in many places. The permutation is daunting, and may be evolutionary evidence for γ-secretase’s initial function in regulated degradation of transmembrane proteins (Kopan and Ilagan, 2004). However, even if γ-secretase was ever simply a “proteasome of the membrane,” its function now is certainly much more complex. As a result, γ-secretase regulation must be at least as intricate and diverse as its function.

γ-Secretase activity is regulated by the assembly of its four essential subunits as well as at the level of the entire complex. Extensive investigation of the former revealed that each of the four essential γ-secretase subunits is tightly and independently controlled. More recently, the importance of regulation of the entire complex has emerged, suggesting additional levels of modulation in γ-secretase function. CD147, phospholipase D1, TMP21, GPR3, γ-secretase activating protein (GSAP), syntaxin-1, Arc, voltage-dependent anion channel 1 (VDAC1), contactin-associated protein 1 (CNTNAP1), TPPP, NDUFS7, Erlin-2, β-arrestin-1, β-arrestin-2, Hif-1α and Nexin 27 have all been implicated as nonessential γ-secretase interacting partners that modulate γ-secretase activity (Zhou et al., 2005; Cai et al., 2006; Chen et al., 2006; Thathiah et al., 2009; He et al., 2010; Teranishi et al., 2010; Wu et al., 2011; Frykman et al., 2012; Hur et al., 2012; Teranishi et al., 2012; Liu et al., 2013; Thathiah et al., 2013; Villa et al., 2014; Wang et al., 2014). However, some of this work is controversial and it remains to be seen whether many of these proteins play a specific and functionally significant role in γ-secretase regulation (Vetrivel et al., 2007, 2008; Hussain et al., 2013). Despite the uncertainty, much can be learned from the way in which a promiscuous enzyme is spatially and temporally modulated by its nonessential cofactors.

We begin by discussing the four essential subunits of γ-secretase, their physiological roles, and, where known, the way in which they regulate γ-secretase activity. Next we move to γ-secretase modulation by nonessential γ-secretase interacting partners, in particular GSAP and Hif-1α, which have been convincingly shown to specifically engage with the active γ-secretase complex. Last we comment on γ-secretase’s regulation by its substrate, APP.

## γ-Secretase is regulated by its four essential subunits

γ-Secretase’s enzymatic promiscuity may on first glance suggest a kind of rampant cleavage activity that indiscriminately chops up anything in its way. Actually, the opposite is true. γ-Secretase may be flexible in its choice of substrate and cleavage site, but its activity is controlled in the cell by a variety of mechanisms, not the least of which is regulation of active complex formation. Perhaps the most basic evidence for this is the finding that γ-secretase activity cannot be increased through the overexpression of PS alone (Levitan et al., 2001), and can be reconstituted only when all four γ-secretase subunits are present (Edbauer et al., 2003). Put another way, the selective ablation of any one of the essential subunits leads to a loss of active complex and enzymatic activity (De Strooper, 2003). This implies that each subunit must be in the proper place and time if there is to be any hope for γ-secretase formation. However, the presence of all four essential subunits does not guarantee active complex formation. This is evidenced by the fact that only a small fraction of steady-state γ-secretase in the cell is actually catalytically active (Beher et al., 2003; Lai et al., 2003; Gu et al., 2004). Furthermore, while overexpression of wild type PS1 in mice is sufficient to increase the amount of γ-secretase complex and protease activity in brains, this is not the case in cellular studies (Li et al., 2011). The implication is that even when all four subunits are in complex with one another, additional events may be necessary to render that complex catalytically active. Some of these events are discussed in the “γ-secretase is regulated by modulatory proteins” section of this review.

The issue is further complicated by the fact that active complexes fall into a wide range of activities with respect to both catalytic efficiency and substrate specificity. Despite the deceptive language used here to describe γ-secretase as “active” and “inactive,” γ-secretase activity is far from a simple on/off switch. Let’s look at the statistics: assuming γ-secretase complexes have a 1:1:1:1 ratio of all essential subunits (Sato et al., 2007), at least four different complexes can be theoretically constructed, keeping in mind the existence of PS and Aph-1 (PS1/PS2 and Aph-1a/Aph-1b) isoforms and the finding that these isoforms do not co-exist in the same complexes (Lai et al., 2003; Shirotani et al., 2004). Identification of the Aph-1a splice variants (Aph-1aS and Aph-1aL) increased the permutation further, to a total of six (Shirotani et al., 2004). Experimentally, different γ-secretase complexes have indeed been identified and shown to vary in catalytic activity (Lai et al., 2003). Some tissue specificity has been observed in the expression of Aph-1 and PS variants, but this alone cannot account for determining which complex gets formed and which does not, especially since different variants of the γ-secretase complex exist dynamically in the same tissue, and even in the same cell line (Placanica et al., 2009a,b). More complicated mechanisms of regulating complex formation pervade, such as the ability of one isoform/mutant to outcompete the other for limiting factors (Placanica et al., 2009a).

The inherent complexity of γ-secretase can be put into perspective by comparing it to signal peptide peptidase (SPP), the only other family of intramembrane aspartyl proteases. Unlike γ-secretase, SPP appears to function alone without the participation of other protein co-factors (Weihofen et al., 2002), although it does form higher order oligomers (Nyborg et al., 2004, 2006; Miyashita et al., 2011). SPP’s simpler structure fits its function as a processor of signal peptides in the membrane, which may not require the same extent of regulation as γ-secretase. Below we discuss each essential γ-secretase subunit individually, paying particular attention to its role in regulating activity.

### Presenilin

PS1 and and its less abundant isoform PS2, are ~50 kDa multipass transmembrane proteins that contain the catalytic core of the γ-secretase complex. These proteins were implicated in γ-secretase function when knock-out of PS1 resulted in severely reduced γ-secretase activity (De Strooper et al., 1998). For a long time it was unclear whether PS contains the active site of γ-secretase or is a chaperone involved in γ-secretase activity or colocalization to substrate. Several critical studies indicated that PS is indeed the catalytic subunit of γ-secretase. First, mutation of the two conserved aspartates in both PS1 (Wolfe et al., 1999) and PS2 (Steiner et al., 1999) significantly reduced Aβ production, suggesting that the aspartates are catalytic or essential residues for γ-secretase activity. Second, aspartyl protease transition-state analogs were shown to directly label and inhibit γ-secretase activity through covalent binding to PS, providing compelling evidence for PS as the catalytic core of γ-secretase (Esler et al., 2000; Li et al., 2000b). Finally, recombinant PS reconstituted into proteoliposomes was shown to be catalytically active even in the absence of other γ-secretase subunits (in contrast to cellular activity which requires all four subunits), providing conclusive evidence for PS’s role as the catalytic subunit of γ-secretase (Ahn et al., 2010).

PS is synthesized as a full length (FL) protein but PS-FL is unstable and is quickly either endoproteolysed or degraded (Ratovitski et al., 1997; Thinakaran et al., 1997; Zhang et al., 1998). Endoproteolysis, a process required for γ-secretase activation, results in the formation of PS1 amino-terminal and carboxy-terminal fragments (PS1-NTF/CTF), which remain associated as a stable heterodimer (Podlisny et al., 1997). In fact, the requirement for endoproteolysis is an onerous one to meet, as evidenced by the fact that in cells it is dependent on the assembly of all four subunits. Furthermore, the ability of FAD mutant PS1ΔE9 (Thinakaran et al., 1996) to be constitutively active despite its inability to be endoproteolysed suggests that evolution has found a way to increase enzymatic activity by circumnavigating an important requirement. A parallel can again be drawn to the PS-like enzyme SPP, which is not endoproteolysed and is active in its FL form, suggesting a simpler form of regulation. The exact molecular mechanism of γ-secretase endoproteolysis remains unclear, but one approach to study it is through the use of active-site directed probes (Gertsik et al., 2014).

As mentioned previously, the formation of an active complex is only the first hurdle in regulating γ-secretase activity in the cell. An added layer of regulation resides in the choice to form PS1 vs. PS2-containing complexes. Different PS variants play different, albeit overlapping, roles as evidenced by genetic knock out studies (De Strooper et al., 1998; Herreman et al., 1999). Later biochemical studies showed that PS1 complexes display a much higher activity than PS2 complexes for a truncated APP substrate (Lai et al., 2003).

PS1 is not only the more active of the two isoforms, but also may be the more amyloidogenic. A reconstitution study in which four γ-secretase isoforms (PS1–aph-1a, PS1–aph-1b, PS2–aph-1a, PS2–aph-1b) were analyzed showed that PS1 complexes form more aggregation-prone Aβ42 (relative to Aβ40) compared to PS2 complexes (Lee et al., 2011). This provides further evidence that the 67% homologous PS isoforms may have different cleavage-site preferences. It would be interesting to investigate whether the PS isoforms also have different substrate preferences. For example, do PS1/PS2 complexes differentially cleave APP/Notch? The decision to form PS1 vs. PS2 complexes probably determines not only “how much” but also “what” is cleaved.

### Pen-2

Pen-2, a ~10 kDa protein with two TMDs (Crystal et al., 2003), was discovered as a gene product that can interfere with PS activity in a genetic study involving *C. elegans* (Francis et al., 2002). Pen-2 is required for endoproteolysis of PS-FL into PS-NTF/CTF and for γ-secretase activity. The following evidence supports that Pen-2 is indispensable for endoproteolysis of PS: first, knock-down of Pen-2 by RNAi resulted in a decrease of PS1-NTF/CTF and a stabilization of the PS1 holoprotein in the Nct-Aph-1 complex, while transient overexpression of Pen-2 in Pen-2 deficient cells led to the recovery of PS fragments (Takasugi et al., 2003). Second, coincorporation of recombinant PS1 and Pen-2 in liposomes showed Pen-2 to be necessary and sufficient for endoproteolysis of PS1 (Ahn et al., 2010). Pen-2 is also required for γ-secretase activity: Pen-2 knockdown in mammalian cells resulted not only in an accumulation of the PS1 holoprotein, but also in a drastic decrease in γ-secretase activity (Takasugi et al., 2003). Pen-2^−/–^ mouse embryos exhibited a Notch-deficiency phenotype and Pen-2^−/–^ MEFs displayed no γ-secretase activity toward APP processing (Bammens et al., 2011). Furthermore, overexpression of human Pen-2 in Pen-2 deficient mice recapitulated AD-like symptoms such as increase in Aβ42, behavioral dysfunctions, and feeding defects, underscoring the importance of Pen-2 in γ-secretase activity and AD pathogenesis (Nam et al., 2011).

The roles of Pen-2 in PS endoproteolysis and γ-secretase activity raised the question—is Pen-2 necessary for γ-secretase activity *per se*, or is Pen-2-inspired endoproteolysis of PS the only requirement for activity? To answer this question the catalytically active PS1ΔE9 endoproteolysis deficient mutant was expressed in Pen-2^−/–^ MEFs and found to have no activity, suggesting that Pen-2 is required for γ-secretase activity *per se*, and not just for endoproteolysis of PS (Bammens et al., 2011). This implies that Pen-2 regulates γ-secretase on multiple levels. We already discussed that Pen-2 dictates activity, as PS-FL is a zymogen that relies on Pen-2-dependent endoproteolysis. This type of regulation is close to being an “on” switch, since lack of WT PS endoproteolysis precludes γ-secretase activity. However, Pen-2 is also capable of more subtle regulation in which it modulates the composition of the γ-secretase complex: overexpression of Pen-2 shifted the equilibrium from PS1 containing complexes to PS2 containing complexes and increased the Aβ42:Aβ40 ratio (Placanica et al., 2009a). Clearly, Pen-2 regulates γ-secretase through a variety of mechanisms, not the least of which are endoproteolysis of PS and complex assembly.

### Nicastrin

In the search for cofactors required for γ-secretase activity, Nct, a type I transmembrane glycoprotein, was the first to be discovered through coimmunoprecipitation with PS1-directed antibody (Yu et al., 2000). A 1.95 Å-resolution crystal structure of Nct from an amoeboid eukaryote *Dictyostelium purpureum* has been solved (Xie et al., 2014). Four hydrophilic residues in the proximal one third of the N-terminal portion of the Nct TMD are critical for interaction between Nct and the rest of the γ-secretase complex (Capell et al., 2003). Nct interacts initially with Aph-1, followed by the incorporation of PS and Pen-2 (LaVoie et al., 2003). Not only does Nct, together with Aph-1, provide a scaffold for the γ-secretase complex, but it also may recognize γ-secretase substrates by binding to their amino termini. Particularly, Nct’s ectodomain has been shown to bind the extracellular regions of APP and Notch after they undergo ectodomain shedding, with Nct’s residues 312–340, and especially Glu333, being most important for substrate recognition (Shah et al., 2005). This finding branded Nct as the substrate-recruiting subunit of γ-secretase. Further confirmation came from work with anti-Nct antibodies: the monoclonal antibody A5226A binds the extracellular domain of Nct causing both a disruption in Nct binding to Notch-based substrate (N100) and a decrease in γ-secretase activity (Hayashi et al., 2011). Additionally, the use of synthetic antibodies showed that a certain structured region in Nct, homologous to the TPR domain involved in peptide recognition, is critical for substrate binding (Zhang et al., 2012). However, several studies called the “substrate-binding” capacity of Nct into question: mutation of mouse Nct-Glu332 (equivalent to human Glu333) to alanine or glutamine was reported to hinder assembly of the γ-secretase complex but not its specific activity, suggesting that substrate recognition/binding was not affected (Chávez-Gutiérrez et al., 2008). Furthermore, Nct-independent, L-685,458 specific, γ-secretase activity has since been detected in two separate MEF lines, suggesting that Nct is in fact not required for substrate recognition (Zhao et al., 2010). These results support Nct’s role in complex assembly and maturation, but not substrate recognition. A subsequent study reexamined the role of Nct and Glu333 in substrate recognition, overturning the previous finding and suggesting once again that Nct is indeed involved in substrate binding (Dries et al., 2009).

While Nct’s role as the substrate-binding subunit of γ-secretase may be controversial, its importance in γ-secretase regulation is uncontested: mutation of two conserved cysteine residues to serine (C213S and C230S) in Nct’s ectodomain resulted in differential γ-secretase processing of APP and Notch in MEF cells lacking endogenous Nct. In particular, APP processing was reduced compared to Notch, suggesting that Nct plays a role in substrate selectivity, although the exact mechanism was not identified (Pamrén et al., 2011). Furthermore, synthetic anti-Nct antibodies were shown to impact substrate selectivity by changing γ-secretase sub-cellular localization (Zhang et al., 2014). Quite possibly Nct regulates γ-secretase activity, and particularly substrate selectivity, through a variety of mechanisms including direct substrate binding, complex formation/stabilization, maturation, and trafficking.

### Aph-1

Aph-1, a ~29 kDa protein with seven TMDs, was discovered in the same genetic screen as Pen-2 (Francis et al., 2002). The GXXXG motif of Aph-1’s TMD4 is crucial for assembly into the γ-secretase complex as it plays a major role in intramembrane helix-helix interactions. Mutation of Gly123 and Gly122 to aspartic acid in *C. elegans* and humans, respectively, results in a loss-of-function phenotype (LOF). The *C. elegans* LOF phenotype gave Aph-1 its name: anterior-pharynx-defective (Goutte et al., 2002). In mammals, mutation of Gly122 to aspartic acid renders Aph-1 incapable of associating with the γ-secretase complex, thereby leading to deficiency in Notch cleavage (Lee et al., 2004).

Humans have two Aph-1 genes that give rise to three versions of the Aph-1 protein (Aph-1aS, Aph-1aL, Aph-1b) due to alternative splicing of the Aph-1a gene (Shirotani et al., 2004). Rodents have an additional isoform, Aph1c, which is a duplication of the Aph-1b gene. Aph-1 isoforms have been reported to differ in their production of longer and shorter Aβ peptides. Particularly, when Aph-1a, Aph-1b, and Aph-1c were individually reintroduced into an Aph1-a^−/–^b^−/–^c^−/–^ mouse, Aph1a rescue of γ-secretase activity resulted in production of shorter Aβ peptides while Aph-1b and Aph-1c rescue led to formation of longer Aβ species. A potential mechanism for this variability may stem from the structural changes evident in PS upon binding to one or the other Aph isoform: fluorescence lifetime imaging microscopy showed that PS may adopt a more closed conformation upon binding to Aph-1b compared to its more open conformation when in complex with Aph-1a (Serneels et al., 2009). This data implies that the choice to incorporate one Aph1 isoform over another can have a profound impact on Aβ production and plaque formation.

## γ-Secretase is regulated by modulatory proteins

γ-Secretase is regulated not only by its four essential subunits, but also by other pathways and proteins, many of which have been identified through LCMS analysis (Teranishi et al., 2010, 2012; Frykman et al., 2012). However, it is unclear whether most of these alleged γ-secretase-interacting partners actually bind the active complex and impact γ-secretase activity. Two γ-secretase-interacting partners, GSAP and Hif-1α, have been reproducibly shown to bind *active* γ-secretase and modulate γ-secretase activity, rendering them both biologically interesting and potentially clinically relevant. Here we describe what is known about the roles these “nonessential” γ-secretase subunits play in regulation.

### GSAP

GSAP is a ~98 kDa holoprotein whose ~16 kDa processed fragment was identified as a γ-secretase-interacting partner. The story began when GSAP was implicated in the mechanism by which imatinib (Gleevec) selectively lowers Aβ without affecting Notch cleavage: a photoactivatable form of imatinib specifically labeled GSAP and not any of the γ-secretase subunits. Further investigation revealed that GSAP may form a complex with γ-secretase and APP-CTF, thereby imparting γ-secretase with substrate specificity for APP over Notch. GSAP RNAi mice crossed with double transgenic APPswe and PS1ΔE9 AD model mice presented a reduced Aβ burden (He et al., 2010). However, a subsequent study questioned GSAP’s role in γ-secretase regulation, reporting that GSAP does not interact with APP-CTF and does not enhance Aβ production in cells or *in vitro* (Hussain et al., 2013). The recent finding that imatinib does in fact regulate GSAP levels and γ-secretase activity supports GSAP’s role as a γ-secretase modulator (Chu et al., 2014). Furthermore, a SNP in GSAP has been found to associate with AD, revealing a new potential risk factor for the disease (Zhu et al., 2014). Taken together, the data provide strong evidence for GSAP’s role in modulating Aβ production, but the precise mechanism by which this occurs needs to be further investigated. GSAP’s function in Aβ formation is the topic of an ongoing debate (Alzforum, 2014).

### Hif-1α

**Figure 3:**
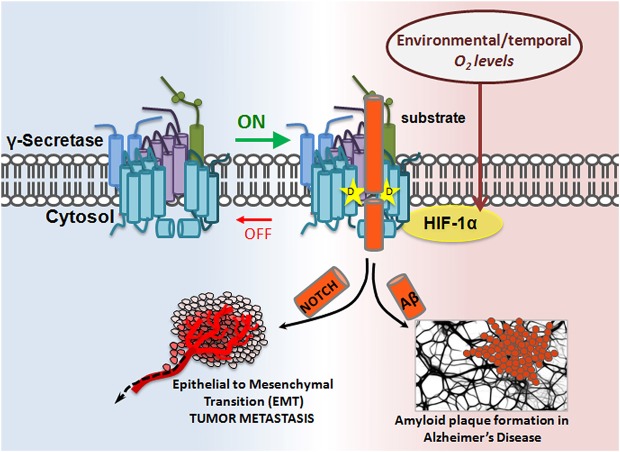
**Modulatory-protein-based regulation of γ-secretase**. Hif-1α, a master regulator of cellular response to hypoxia, is a γ-secretase interacting partner. In the presence of sufficient oxygen, no Hif-1α is produced. However, in oxygen-deficient conditions, Hif-1α is upregulated. Hif-1α binding to γ-secretase enhances γ-secretase activity by increasing the ratio of active:inactive γ-secretase complexes in the cell, thereby increasing γ-secretase cleavage of its substrates, such as APP and Notch. The implication is twofold: first, inactive γ-secretase complexes are physiologically important, and second, γ-secretase can be temporally regulated.

Hif-1α, a ~93 kDa protein, is a master regulator of cellular response to hypoxia. While Hif-1α’s properties as a transcription factor and role in Notch signaling are well documented (Gustafsson et al., 2005; Mukherjee et al., 2011; Wang et al., 2011), its role in γ-secretase regulation was recently discovered: Hif-1α directly binds to γ-secretase, stimulating its activity for Notch cleavage. Data show that hypoxic conditions enhance γ-secretase cleavage of Notch in a Hif-1α-dependent manner. Furthermore, it appears that the mechanism for γ-secretase activation is through a shift in equilibrium from inactive to active complexes upon Hif-1α binding, suggesting that Hif-1α has the capacity to turn an inactive γ-secretase into an active one (Villa et al., 2014). This is a common form of enzymatic regulation in which the enzyme is abundant in the cell in its “off” state and can be quickly mobilized should an “on” signal appear. Hif-1α studies presented the first evidence for the cell’s ability to activate γ-secretase complexes that were previously inactive, and for γ-secretase’s subjugation to temporal regulation (Figure 3).

## γ-Secretase is regulated via its substrate, APP

**Figure 4:**
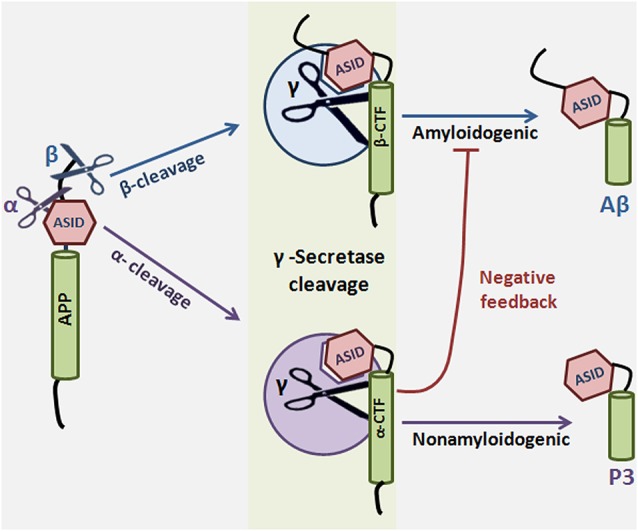
**Substrate-based regulation of γ-Secretase**. α or β-secretase cleave amyloid precursor protein to generate αCTF or βCTF, respectively. αCTF and βCTF, which are γ-secretase substrates, can then interact with γ-secretase to form a substrate-enzyme complex. The substrate inhibitory domain (ASID) in αCTF and βCTF interacts with γ-secretase in a location distinct from both the active site and the substrate binding site of the enzyme. Binding of αCTF to γ-secretase results in a low-productivity complex: the ASID in αCTF inhibits γ-secretase activity for Aβ production. ASID in βCTF is a less potent inhibitor of γ-secretase. The implication is that α-secretase cleavage potentiates γ-secretase inhibition by at least two mechanisms: first, α-secretase cleaves APP in the middle of the Aβ region, precluding Aβ formation, and second, α-secretase cleavage results in formation of αCTF, which inhibits γ-secretase activity through its ASID. γ-Secretase cleavage of αCTF produces p3 and cleavage of βCTF produces the amyloidogenic Aβ.

APP proteolysis falls into one of two pathways: either APP is cleaved sequentially by α and γ-secretase in the nonamyloidogenic pathway, or it is cleaved by β and γ-secretase in the amyloidogenic pathway. α-Secretase cleavage of APP occurs in the Aβ region, which precludes Aβ production, so the choice to preferentially undergo more α cleavage can mean the difference between healthy and disease states. What factors determine the decision to participate in one pathway over the other? It has been suggested that α-secretase competes with β-secretase for the APP substrate, thereby lowering Aβ formation (Lammich et al., 1999; Skovronsky et al., 2000; Postina et al., 2004). However, compelling evidence exists for an alternate mechanism in which α-secretase cleavage results in formation of a substrate inhibitory domain (ASID) within αCTF that binds to an allosteric site in γ-secretase, thereby inhibiting γ-secretase processing of βCTF and ultimate Aβ production. In this model, α-secretase plays a dual anti-amyloidogenic role: first, it cleaves APP in the Aβ region, thereby directly precluding Aβ formation, and second, it initiates a feedback loop in which αCTF binds γ-secretase and acts as a γ-secretase modulator which specifically lowers Aβ production (Tian et al., 2010b). ASID is also present in βCTF, suggesting that the product of β-secretase cleavage is imbued with γ-secretase-regulating capacity as well. The Flemish FAD mutation, located in the ASID domain, interferes with βCTF’s inhibitory potency, leading to increased Aβ (Tian et al., 2010a). ASID is the first example of a substrate’s inherent ability to regulate γ-secretase, but it is probably not the last (reviewed in Zhang and Xu, 2010) (Figure 4).

## Conclusion

γ-Secretase is regulated at many levels, including but not limited to regulation by “essential” subunits, complex formation, “nonessential” subunits, substrates, and lipid composition (Holmes et al., 2012; Walter and Van Echten-Deckert, 2013). Although the importance of PS in γ-secretase activity is well established, it appears that the other essential subunits also notably contribute to regulating activity and substrate specificity beyond just their roles in complex assembly. Furthermore, nonessential subunits like GSAP and Hif-1α fine-tune modulation of the already stringently-regulated enzyme, thereby presenting potential therapeutic opportunities for modulating γ-secretase activity without the mechanism-based toxicities that result from γ-secretase inhibition (i.e., GSIs).

Currently, GSMs are being developed as a promising treatment for AD (Crump et al., 2013) and animal studies indicate that they could provide advantages over GSIs (Mitani et al., 2012; Rogers et al., 2012). Other Aβ-based therapies, such as immunotherapy and autophagic restoration, are being explored for AD as well (Lemere, 2013; Li et al., 2013). In addition to mice (Eimer and Vassar, 2013; Veeraraghavalu et al., 2013), other models have been investigated for AD studies (Mccoll et al., 2012; Do Carmo and Cuello, 2013), which help to elucidate the role of γ-secretase in pathogenesis. Recently, it has been suggested that a set of γ-secretase product peptides in the cerebrospinal fluid may serve as biomarkers for AD (Hata et al., 2012; Rosén et al., 2013). No doubt, developing a better understanding of γ-secretase in AD pathogenesis will facilitate the discovery of effective biomarkers and safe treatments. The complex levels of γ-secretase regulation that are now emerging may improve our ability to develop targeted therapies for AD and cancer.

## Conflict of interest statement

The authors declare that the research was conducted in the absence of any commercial or financial relationships that could be construed as a potential conflict of interest.
